# Photodynamic Therapy in Ocular Oncology

**DOI:** 10.18502/jovr.v15i4.7793

**Published:** 2020-10-25

**Authors:** Mehdi Mazloumi, Lauren A Dalvin, Seyed-Hossein Abtahi, Negin Yavari, Antonio Yaghy, Arman Mashayekhi, Jerry A Shields, Carol L Shields

**Affiliations:** ^1^Ocular Oncology Service, Wills Eye Hospital, Thomas Jefferson University, Philadelphia, USA; ^2^Department of Ophthalmology, Mayo Clinic, Rochester, USA; ^3^Isfahan Eye Research Center, Feiz Hospital, Isfahan University of Medical Sciences, Isfahan, Iran; ^4^Department of Cardiovascular Research, Tehran Heart Center, Tehran University of Medical Sciences, Tehran, Iran

**Keywords:** Choroid; Eye, Hemangioma, Melanoma, Metastasis, Photodynamic therapy Retina, Tumor

## Abstract

Over the past two decades, we have witnessed the increasing use of photodynamic therapy (PDT) in the field of ocular oncology. Based on a review of the literature and our own experience, we herein review the role of PDT for the management of intraocular tumors. The discussion includes two main topics. First, we discuss the application of PDT for benign tumors, including circumscribed choroidal hemangioma, choroidal osteoma, retinal astrocytoma, retinal capillary hemangioma (retinal hemangioblastoma), and retinal vasoproliferative tumor. Second, we assess the role of PDT for malignant tumors, including choroidal melanoma and choroidal metastasis.

##  INTRODUCTION

Photodynamic therapy (PDT) is a form of laser therapy that targets abnormal capillaries and has been useful for the treatment of intraocular neovascularizations and neoplasms.^[1,2]^ The technique of PDT involves the intravenous administration of a photosensitizing chemical substance, currently verteporfin, followed by targeted application of a low power and long duration infrared laser beam. Activation of verteporfin by the laser causes formation of free radicals, which in turn leads to damage to the leaking blood vessels, resulting in the closure of the vessels and resorption of the related fluid. In the ophthalmic field, PDT was initially conceived as a therapy for macular choroidal neovascularization in eyes with age-related macular degeneration.^[3]^ Later, PDT was employed for polypoidal choroidal vasculopathy, central serous chorioretinopathy, and other retinal conditions.^[4,5]^ PDT has been also used as therapy for selected intraocular tumors.

PDT acts through two mechanisms with regard to intraocular tumors: (1) direct tumor destruction via selective cytotoxic activity against tumor cells, and (2) through the promotion of intraluminal photothrombosis in the vessels supplying the tumor.^[6]^ In this review, we present a summary on the role of PDT in the management of various benign and malignant intraocular neoplasms.

##  METHODS

A comprehensive literature search was performed using the PubMed and Scopus databases for English-language publications using the keywords “photodynamic therapy”, “circumscribed choroidal hemangioma”, “choroidal osteoma”, “ retinal astrocytoma”, “retinal capillary hemangioma”, “retinal hemangioblastoma”, “ retinal vasoproliferative tumor”, “ choroidal melanoma”, “choroidal metastasis”, “intraocular tumors”, and “ocular oncology” for all papers published from January 2002, the time when PDT became available for use in the ophthalmic field, to May 2020. The relevant articles were reviewed, and key findings were extracted.

### PDT Methods Used in Ocular Oncology

Verteporfin (VisudyneⓇ Novartis International AG, Basel, Switzerland) with a total dose of 6 mg/m${}^{2}$ is injected intravenously slowly over 10 min and accumulates in the retinal, choroidal, and tumor vasculature. The following calculations can be performed to appropriately reconstitute verteporfin at the correct dosage for a patient based on body surface area (BSA):

• BSA = $\frac{\sqrt{\mathrm{Height}(\mathrm{in})\times \mathrm{Weight}(\mathrm{lbs})}}{3131}$
• Total drug dose = 6 mg/m${}^{2}$ × BSA
• Volume of reconstituted verteporfin = Total drug dose ÷ 2.0 mg/mL
• Volume of dextrose 5% in water (D5W) = 30 mL – volume of reconstituted verteporfin

The infrared laser beam (Coherent Opal Photoactivator Diode Laser; Coherent Inc., Santa Clara, CA, USA) is then applied 5 min after the completion of infusion with the following properties (standard fluence):

• Wavelength: 689 nm
• Radiant exposure: 50 J/cm${}^{2}$ for 83 s (Irradiance: 600 mW/cm${}^{2}$)

In some cases, the half-fluence method is used to deliver 25 J/cm2 of energy and is usually utilized for more photosensitive tumors.

While in some cases, double-dose method is used to deliver 100 J/cm${}^{2}$ by administering a total verteporfin dose of 12 mg/m${}^{2}$, in some cases, the method is used to deliver 100 J/cm${}^{2}$ by applying laser for 166 s.^[1,2]^

##  RESULTS

### Indications for PDT in Ocular Oncology

#### Part A: Benign Tumors

#### A.1: Benign Choroidal Tumors

#### I. Circumscribed choroidal hemangioma

**Figure 1:**
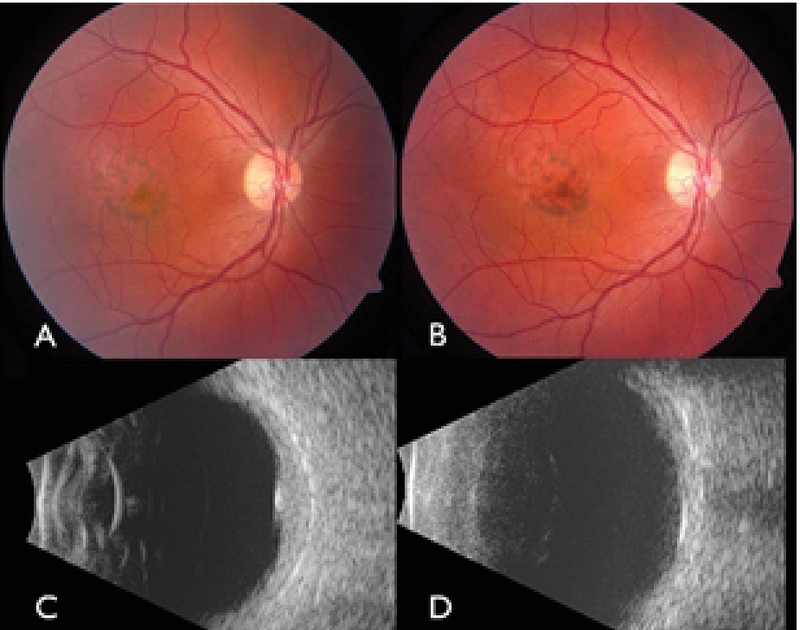
Fundus photograph of the right eye of a patient with choroidal hemangioma located in the macula (A) before and (B) three months after photodynamic therapy (PDT). B-scan ultrasonography (C) before and (D) after PDT revealed a decrease in tumor thickness.

Circumscribed choroidal hemangioma (CCH) is a benign vascular hamartoma, frequently diagnosed as a solitary, orange-red, dome-shaped mass in the posterior pole.^[7]^ Patients aged <20 years present with worse visual acuity and larger, more posterior tumors.^[8]^ When the lesion is asymptomatic, usually as an extrafoveal mass detected in routine retinal examination, observation alone is appropriate. In cases with visual distortion or reduced visual acuity, due to a foveal or juxtafoveal tumor causing cystoid macular edema (CME) or subretinal fluid (SRF), intervention can improve the long-term visual acuity outcomes.^[9]^ Due to the risk of retinal scarring and other ocular complications associated with radiation therapy, laser photocoagulation, and thermotherapy, these modalities are less favored as primary treatment.^[10]^ PDT, in contrast, spares the overlying retina and has efficacy in both reducing CCH thickness and causing resolution of associated serous SRF, resulting in stability or improvement of visual acuity (Figure 1).^[11,12,13,14,15]^ Shields et al, in a series of 458 cases, found that the management of CCH in the PDT era has allowed for improved visual acuity outcomes compared with the pre-PDT era, with mean final visual acuity of 20/63 (PDT era) versus 20/400 (pre-PDT era).^[16]^ Ho et al reported that patients presenting before the age of 50 years with pretreatment best-corrected visual acuity (BCVA) ≥ 20/200 and less foveal edema were most likely to benefit from PDT.^[17]^ In a recent study by Di Nicola et al, investigating the predictive factors of visual outcome in 79 patients with CCH treated with PDT, the authors found correlations between good final visual outcome (≥20/40) and good baseline visual acuity, smaller tumor size, lack of CME, and lack of treatment prior to PDT.^[18]^

**Figure 2:**
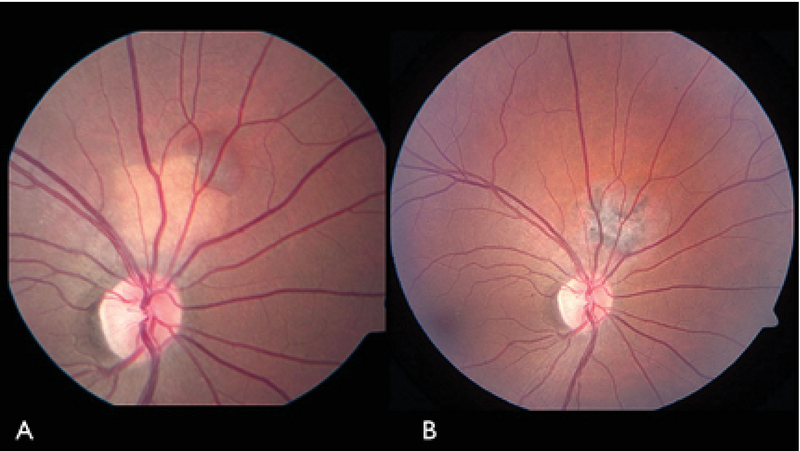
Fundus photograph of the right eye of a patient with juxtapapillary choroidal osteoma associated with subretinal hemorrhage (A) before and (B) after PDT. PDT resulted in complete regression of the tumor and resolution of subretinal hemorrhage.

**Figure 3:**
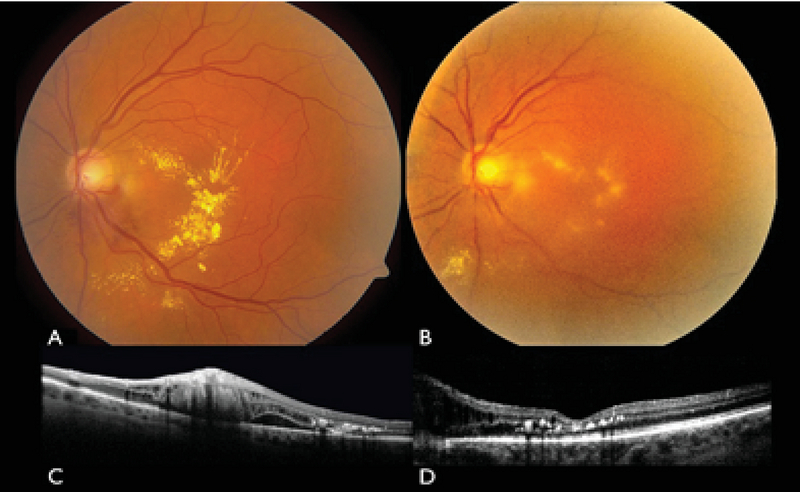
Fundus photograph of the left eye of a patient with juxtapapillary retinal hemangioblastoma associated with macular edema and subretinal fluid and lipid exudation (A) before and (B) 20 months after PDT. PDT resulted in the regression of the tumor and the resolution of the macular edema confirmed by optical coherence tomography (OCT) (C, D).

**Figure 4:**
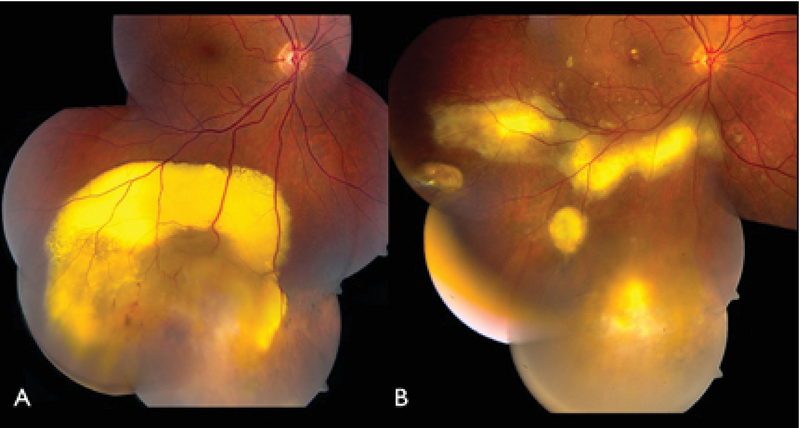
Fundus photograph of the right eye of a patient with a vasoproliferative tumor located inferiorly (A) before and (B) 18 months after PDT.

**Figure 5:**
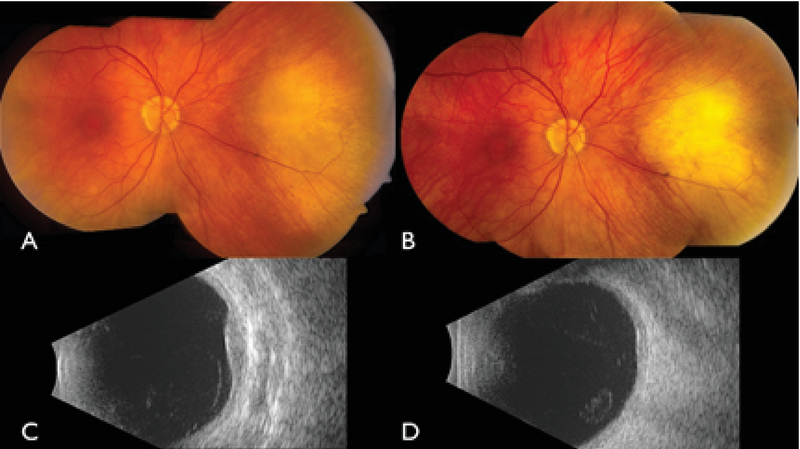
Fundus photograph of the right eye of a patient with amelanotic choroidal melanoma (A) before and (B) after PDT. PDT resulted in the regression of the tumor confirmed with B-scan ultrasonography (C, D).

**Figure 6:**
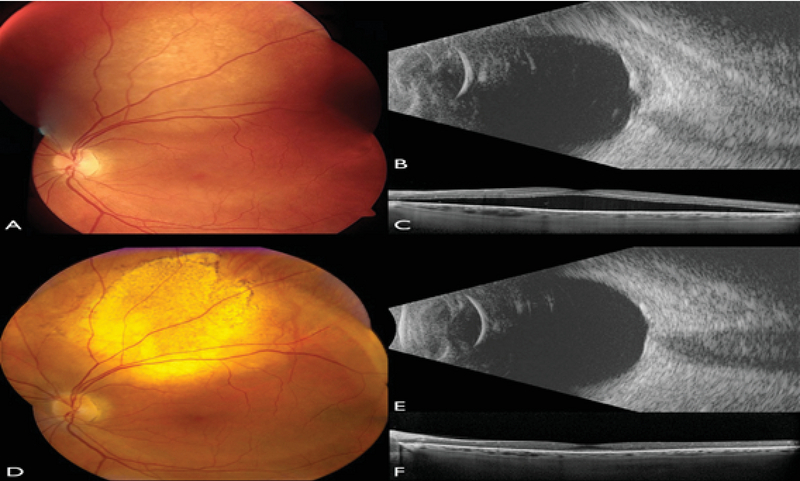
Choroidal metastasis managed with PDT. Fundus photograph of the left eye of a patient with choroidal metastasis located superior to the macula (A) and confirmed by B-scan ultrasonography (B). The tumor was associated with subfoveal fluid as seen on OCT (C). Following the PDT application, there was a complete tumor regression (D) confirmed by B-scan ultrasonography (E) and resolution of the subfoveal fluid as seen on OCT (F).

With regards to the treatment parameters, double-dose PDT (12 mg/m${}^{2}$) provided better tumor regression with similar resorption of SRF compared with standard-dose PDT (6 mg/ m${}^{2}$). Single-spot PDT was as effective and safe as overlapping-spot PDT (two to three spots).^[19,20]^ Moreover, although bolus PDT (6 mg/m${}^{2}$ verteporfin infusion bolus in 1 min; treatment at 5 min; 100 J/cm${}^{2}$; 166 s) is capable of inducing tumor regression, the resulting retinal pigment epithelium (RPE) and retinal changes can lead to reduced visual acuity.^[21]^ Some clinicians have postulated that patients who require multiple treatment sessions might experience recurrent leakage at more frequent intervals.^[22]^

#### II. Choroidal osteoma 

Choroidal osteoma is a benign, ossifying tumor of unknown origin that often appears as a unilateral, orange–yellow choroidal mass with well-defined or scalloped borders located in the juxtapapillary or macular region of young women in their early twenties.^[23,24]^ In a series of 74 eyes with choroidal osteoma reported by Shields et al, the authors found evidence of growth in 51% of eyes and decalcification in nearly 50% of eyes with poor visual acuity of 20/200 or worse in 56% of eyes by 10 years.^[25]^ Although benign, choroidal osteoma can result in significant visual acuity loss by profound outer retinal layer thinning and photoreceptor loss over the subfoveal portion of the tumor.^[26]^

Shields et al were the first to report the beneficial effect of PDT on extrafoveal choroidal osteoma. In their report, PDT was applied to the entire juxtapapillary choroidal osteoma with an overlying subretinal hemorrhage using a single 83-s laser spot at 689 nm (50 J/cm2). The hemorrhage resolved by one month and the complete regression of the osteoma was noted after nine months of follow-up (Figure 2 ).^[27]^ Mazloumi et al recently evaluated the efficacy of PDT in nine eyes of nine patients with extrafoveolar choroidal osteoma and found complete (4/9, 44%) and partial (5/9, 56%) tumor regression with a mean of 73% regression in the PDT-treated areas after 49 months of follow-up, which was significantly greater than the spontaneous regression rate of 28% previously reported at a five-year follow-up. They concluded that PDT is a valuable modality for the management of extrafoveal choroidal osteoma, with the intent to decalcify and involute the tumor so that further tumor growth under the foveola would be prohibited and visual acuity would be preserved. They also cautioned that PDT should not be employed for subfoveal choroidal osteoma as this could lead to choroidal atrophy and secondary photoreceptor retraction in the central macular area with poor visual outcome.^[28]^

#### A.2: Benign Retinal Tumors

#### III. Retinal astrocytoma 

Benign retinal astrocytic tumors are comprised of three different entities: astrocytic hamartoma, acquired astrocytoma, and reactive retinal gliosis. Astrocytic hamartoma, which is most frequently detected in children with tuberous sclerosis complex or neurofibromatosis, is typically a stable tumor.^[29]^ However, acquired astrocytoma, which is most frequently found in young or middle-aged adults without tuberous sclerosis complex, is a sporadic tumor typically located in the macular or juxtapapillary region. Acquired astrocytoma typically presents with abundant intrinsic vascularity, slow progressive growth, and exudation that can cause visual acuity loss and even lead to total exudative retinal detachment necessitating enucleation.^[30,31,32,33,34,35]^ PDT, plaque radiotherapy, external beam radiotherapy, laser photocoagulation, endoresection, and enucleation have been used to control retinal astrocytoma with limited reports on their beneficial effects.^[36,37,38,39]^

Mennel et al reported a case of an exudative astrocytic hamartoma in a patient with tuberous sclerosis treated with PDT. Tumor size reduction along with the resolution of SRF and improvement in visual acuity were observed after treatment.^[40]^ In 2008, Shields et al successfully applied PDT in a patient with retinal astrocytoma and associated macular exudation, edema, SRF, and decreased visual acuity to 20/70, which was unresponsive to laser photocoagulation.^[31]^ After the treatment, the resolution of macular exudation, edema, and SRF were noted, resulting in improved visual acuity initially of 20/50 (at one month) and ultimately 20/30 (at 4, 8, and 12 months).^[31]^ House and colleagues, in 2016, reported successful treatment of a juxtapapillary retinal astrocytoma with one session of PDT in a 50-year-old man with a visual acuity of 20/100. Treatment resulted in complete resolution of surrounding SRF and lipid exudation. At the 20 months follow-up, the visual acuity improved to 20/20 with complete tumor regression and normal foveal contour on optical coherence tomography.^[41]^ Eskelin et al reported two cases of aggressive retinal astrocytoma with secondary lipid exudation and exudative retinal detachment successfully treated with a single session of PDT.^[34]^ The growing vascularized portion of both tumors regressed, and the exudative retinal detachment completely resolved. Regression was associated with obliteration of the intrinsic vessels within the growing part of the tumors as well as closure of the dilated retinal capillaries over the tumors.^[34]^ The authors proposed PDT as a first-line treatment for aggressive retinal astrocytoma.

#### IV. Retinal capillary hemangioma (retinal hemangioblastoma)

Genetically, retinal capillary hemangioma (RCH) can be found either as an isolated lesion or as part of the von Hippel–Lindau (VHL) syndrome. In a recent study, the VHL syndrome was the underlying cause of RCH in 84% of cases, more often than previously reported.^[42]^ Hence, genetic and clinical VHL screening should be performed in all patients with RCH. Phenotypically, RCH can either be juxtapapillary or located elsewhere in the retina (extrapapillary). Extrapapillary RCH usually starts as a tiny red intraretinal lesion, measuring less than a few hundred microns in diameter. With increasing size, RCH might manifest more distinctive features, including increasing nodularity, feeding and draining blood vessels that become progressively dilated and tortuous, and exudative retinopathy.^[43]^ Fluorescein angiography (FA) is the best diagnostic modality for the detection and confirmation of RCH because FA shows rapid filling of the feeding artery, then the tumor, followed by the rapid exit through the draining vein. More importantly, subclinical pinpoint tumors can be detected on FA before they become symptomatic.^[44]^ Disease progression can be devastating because exudation from the vascular tissue can affect the macula and cause glial proliferation and tractional retinal changes.

The management of RCH is debated and the feasibility and success of treatment depend on several factors, including size and location of the tumor, severity of exudation, associated retinal detachment, and epiretinal fibrosis or hemorrhage.^[45,46,47]^ Tumors associated with VHL syndrome tend to have more aggressive behavior. Therefore, nearly all RCHs must be considered for the treatment. If lesions are small (<2 mm) in size, laser photocoagulation or PDT can be applied; if medium (2–4.5 mm), PDT or cryotherapy can be used; and if large (>4.5 mm), cryotherapy, brachytherapy, or endoresection might be employed.^[44]^ There may also be a beneficial role for injections of intravitreal anti-vascular endothelial growth factor (VEGF) agents.^[48]^

PDT has been used successfully for the management of RCH.^[49,50]^ In a series of six eyes reported by Sachdeva et al, PDT resulted in tumor regression or stabilization and improvement of SRF and lipid exudation in all cases.^[51]^ However, stabilization or improvement of visual acuity was noted in only 50% of the cases. The authors stated that the benefits of PDT might be limited by pre-existing macular changes and progression of epiretinal membrane.^[51]^ In a series of ﬁve eyes with RCH (four juxtapapillary and one extrapapillary tumors) by Papastefanou et al, two different PDT treatment protocols were employed for eyes with juxtapapillary RCH, that is, two eyes received double-duration, full-ﬂuence PDT, while the other two eyes received single-duration, half-ﬂuence PDT. This variable PDT protocol did not affect the treatment outcome as one eye in each group showed partial resolution of macular edema. Despite the improvement in macular edema in 50% of the patients with juxtapapillary RCH, this improvement did not result in an improvement in visual acuity after treatment.^[52]^ However, the extrapapillary RCH in this series showed regression of the tumor and macular edema with VA improvement, suggesting a better anatomical and functional outcome following the PDT application for peripheral tumors.^[52]^ The authors stated that although peripheral RCH is presumed to be more amenable to the treatment due to its peripheral location, complications including epiretinal membrane formation and tractional RD can occur.^[52]^

We believe that PDT should be considered as a nonablative modality for the treatment of RCHs that <4.5 mm in diameter (Figure 3). Extrapapillary RCH >4.5 mm in diameter pose a difficult challenge and PDT may be ineffective for these tumors.^[45]^

#### V. Retinal vasoproliferative tumors 

Retinal vasoproliferative tumors (VPT) were first described in 12 patients in 1983 by Shields et al,^[53]^ and later, the same group provided clinical descriptions on 103 cases in 1995 ^[54]^ and 334 cases in 2013,^[55]^ further improving our understanding of this uncommon retinal tumor.

VPT is a reactionary glial cell proliferation with secondary vasoproliferation presenting as a vascular nodular tumor arising in the neurosensory retina with associated telangiectasia, lipid exudation, and SRF.^[56]^ While three-quarters are primary and isolated, roughly a quarter of such lesions are secondary to various pre-existing inflammatory, infectious, congenital, iatrogenic, hereditary, or traumatic retinal conditions.^[54,57]^ VPT is less frequently observed in the posterior pole and has a predilection for the retinal periphery, especially the inferotemporal quadrant.^[58]^ These lesions can be sight-threatening by causing lipid exudation with or without exudative retinal detachment, preretinal fibrosis, intra- and subretinal hemorrhage, RPE proliferation, epiretinal membrane, and CME.^[54]^

Management options include observation, laser photocoagulation, cryotherapy, PDT, and plaque radiotherapy. Asymptomatic and isolated small, peripheral VPTs with minimal exudation posing no visual threat are best managed with cautious observation. Treatment is necessary for tumors that threaten or affect visual acuity due to progressive exudation. Smaller tumors can be managed with laser photocoagulation, PDT, or cryotherapy, while larger tumors (>2–3 mm) can be managed with plaque radiotherapy with reported tumor regression in >90% of cases.^[59,60]^ Intravitreal anti-VEGF medications have been recently used in few case reports with promising results. However, the effect seems to be short-lived and recurrence of exudation can occur following the cessation of treatment.^[61,62]^

Application of PDT in the peripheral retina may be technically challenging, and there is much less experience on its successful use for the treatment of VPT.^[63]^ PDT can be applied as single or multiple spots depending on the size of the lesion (Figure 4). Hussain et al in a series of 25 eyes with VPT treated with PDT found the resolution of exudation in 76% (19/25), regression of the tumor in 76% (19/25), and visual gain or stabilization in 92% (23/25).^[64]^ The authors noted a decrease in the success rate in tumors with extensive exudation, as only one-half of the cases with exudative retinopathy achieved an adequate response.^[64]^ Blasi et al reported successful treatment of VPT with one session of PDT in three patients. They observed neither recurrence nor complications at a one-year follow-up.^[65]^ In another study by Barbazetto et al, a 47-year old patient developed a secondary VPT and excessive exudation secondary to scleral buckling surgery. Two different light doses (50 J/cm2 and 100 J/cm2) were applied in two consecutive PDT sessions, and the tumor became fibrotic and exhibited no sign of vascularity or leakage after the treatment.^[56]^

#### Part B: Malignant Tumors

#### I. Choroidal Melanoma

Choroidal melanoma is the most common primary intraocular malignancy of adulthood, which appears as an amelanotic-to-solid brown, dome-shaped, or mushroom-shaped mass.^[66,67]^ Choroidal melanoma usually occurs in Caucasians with no sex predominance, and in contrast to cutaneous melanoma, ultraviolet radiation exposure plays no known role in its pathogenesis.^[68]^ There is no strong familial inheritance pattern for this neoplasm in most patients, although some cases may be associated with the BAP1 tumor predisposition syndrome.^[69]^ Shields et al in a large survey on 8,033 cases investigating clinical spectrum and prognosis of uveal melanoma found that there were 106 (1%) cases in young patients (≤20 years), 4,287 (53%) cases in mid adults (21–60 years), and 3,640 (45%) cases in older adults (>60 years).^[70]^ The overall mortality rate of choroidal melanoma in their report was as high as 20% at 20 years, mainly due to metastasis to the liver.^[70]^ Prognosis of choroidal melanoma is dependent on several factors, including size and location of the tumor, extrascleral extension, and tumor cytogenetics.^[71,72]^

The management of choroidal melanoma involves careful evaluation of tumor size and location, with systemic workup for distant metastasis. For large tumors, enucleation may be required.^[73]^ Globe-sparing methods can be used for the treatment of small, medium, and some large choroidal melanomas, including radiotherapy (plaque, proton-beam, gamma knife, or stereotactic), transpupillary thermotherapy (TTT), and PDT.^[74,75,76]^ Plaque radiotherapy is currently the treatment of choice in most cases with local tumor control rates of as high as 97% and minimal damage to surrounding orbital tissues. However, damage to the retina, optic nerve, and anterior structures of the globe can occur in many cases despite adequate tumor control.^[77]^ Photocoagulation has been used in the past for the treatment of small choroidal melanomas but is associated with relatively high rates of local treatment failure and delayed local tumor recurrence.^[78]^ Non-coagulative laser therapy, referred to as TTT was commonly used for the treatment of small choroidal melanomas. Mashayekhi et al in a study of small choroidal melanomas treated between 2001 and 2012 with TTT, found a Kaplan–Meier estimate for tumor recurrence of 11% at 5 years and 15% at 10 years.^[76]^ The authors advised that, when possible, small choroidal melanomas with multiple risk factors should be treated with methods other than TTT. Currently, TTT is typically reserved for small choroidal melanomas with one or two risk factors or as supplementary treatment following plaque radiotherapy or proton beam irradiation.^[76,79]^

PDT is a convenient, cost-effective, well-tolerated option for outpatient settings and, in contrast to TTT, is painless at the time of application.^[80]^ In a study on 12 amelanotic or lightly pigmented small choroidal melanomas managed with PDT, Turkoglu et al found complete tumor regression after one (*n* = 3, 25%), two (*n* = 3, 25%), and three (*n* = 2, 17%) sessions of primary PDT, with a stable or improved visual acuity (Figure 5).^[74]^ Campbell et al in another study on nine patients with posteriorly located amelanotic choroidal melanomas (one with a pigmented portion) found complete tumor regression in eight amelanotic cases. Although the amelanotic portion of the mixed tumor flattened, the height of the pigmented part remained stable.^[80]^ Barbazetto et al conducted a study on four patients with choroidal melanoma who had local failure following plaque radiotherapy and TTT. After the secondary PDT application, two eyes were salvaged and two melanomas continued to grow, necessitating enucleation.^[81]^

It is believed that for PDT to be effective, choroidal melanoma should be non-pigmented or minimally pigmented to allow penetration of the laser light to the intrinsic tumor vessels. However, Fabian et al in a study on 15 patients with small pigmented posterior pole choroidal melanoma who were treated with three sessions of PDT found tumor control in 12 (80%) patients at 15 months follow-up. Of note, all the three (20%) failed cases were 100% pigmented, de novo melanomas rather than transformed nevi, and showed a radial growth pattern rather than increased thickness.^[82]^ It has been claimed that the presence of SRF may be a harbinger of improved response to PDT due to the presence of underlying leaking vessels.^[83,84]^ Although PDT of small choroidal melanomas is associated with a lower rate of tumor control (80–89%) compared with radiotherapy (95–97%), the visual acuity can be maintained or improved after PDT compared with an increased possibility of vision loss following radiotherapy modalities.^[83]^

Some researchers have evaluated the efficacy of the combination of PDT with radiotherapy. Blasi et al in a study on 26 patients have shown that PDT as neoadjuvant therapy before plaque radiotherapy reduced tumor thickness in 73% of cases, thereby decreasing the necessitated dose of radiation for subsequent radiotherapy without compromising disease control.^[85]^ Tuncer et al reported a patient with an amelanotic choroidal melanoma of 6.5 mm thickness who showed poor response to Iodine plaque radiotherapy (80 Gy apical dose) with no reduction in thickness at 16 months follow-up. The authors documented dramatic tumor regression over two months to a completely flat scar (1.3 mm thickness) following the application of PDT using three overlapping spots.^[86]^ The authors hypothesized that primary radiotherapy might cause better "recirculation and re-oxygenation" permitting improved concentration of photoactive dye in the tumor vasculature for subsequent PDT.

#### II. Choroidal metastasis 

Choroidal metastasis is the most common intraocular malignancy in adults, typically appearing as a solitary yellow mass with associated SRF.^[87,88]^ In a large survey on 2,214 uveal metastasis by Shields and colleagues, the primary tumor originated in the breast (37%), lung (26%), kidney (4%), gastrointestinal (GI) tract (4%), cutaneous melanoma (2%), lung carcinoid (2%), prostate (2%), thyroid (1%), pancreas (1%), other sites (3%), and unknown (16%). The worst survival was found in patients with pancreatic metastasis (mean 4.2 months) and the best survival with lung carcinoid (92% at 5 years).^[89]^ Choroidal involvement may occur at any stage of the primary malignancy, but those that tend to present in the late course of malignancy are associated with a worse prognosis.^[88,90]^

In the literature, several options have been proposed for the management of choroidal metastatic lesions based on the size, location, number of metastatic tumors, systemic status, and laterality.^[88]^ Observation, systemic chemotherapy, radiotherapy, TTT, and PDT are current treatment strategies. PDT is a safe, non-invasive procedure, and the highly vascular nature of choroidal metastatic tumors makes them amenable to PDT.

Several case reports and series have been published on the use of PDT in the management of choroidal metastasis, with acceptable results. In a recent retrospective interventional case series of 40 eyes with 58 choroidal metastatic tumors, PDT showed promising results, achieving tumor control with one (*n* = 32 tumors [71%]) or two (*n* = 3 tumors [7%]) sessions (Figure 6). The study showed that the primary cancer site or ocular tumor features (size, location, color, shape, related SRF) did not impact tumor control.^[90]^ In a series by Kaliki et al consisting of nine metastatic lesions in eight eyes, regression of the tumor was documented in seven tumors (78%) and stabilized or improved vision was noted in seven eyes (88%).^[91]^ In another series by Ghodasra et al, 17 of 21 tumors (81%) were flat at 12 months follow-up and 18 tumors (86%) showed complete resolution of SRF.^[92]^

##  DISCUSSION

PDT is a well-tolerated outpatient modality for the treatment of selected benign or malignant intraocular tumors. Over the past two decades, our knowledge of the potential role of PDT in the field of ocular oncology has increased substantially but further studies are needed to explore the full potential and limitations of this relatively novel therapeutic modality.

##  Financial Support and Sponsorship

This review has been supported in part by the Eye Tumor Research Foundation, Philadelphia, PA (JAS, CLS). The funders had no role in the design and conduct of the study, in the collection, analysis, and interpretation of the data, and in the preparation, review, or approval of the manuscript.

##  Conflicts of Interest

There are no conflicts of interest.
